# Southern Dental Association

**Published:** 1885-07

**Authors:** 


### SOUTHERN DENTAL ASSOCIATION.

SEVENTEENTH ANNUAL MEETING, AT NEW ORLEANS.

REPORTED FOR THE INDEPENDENT PRACTITIONER, BY MRS. M. W. J.

(Continued from page 313.)

#### SECOND DAY—AFTERNOON SESSION.

The subject of Pathology and Therapeutics was taken up, and the paper of the President, Dr. A. O. Rawls, on Pyorrhœa Alveolaris, was discussed. The following is a brief *resume* of the paper:

After enumerating the different theories of causation, local and systemic, salivary and seruminal calculi, fungi, bacteriæ, etc., the symptoms of the disease were minutely described. The first subjective symptom was pronounced a sense of fullness or of impactedness, followed by a soreness or an elastic feeling of the gums under percussion; then follows a slight thickening or prominence, accompanied by a bluish tint, indicating increased size of capillaries, a disruption of the tissues being succeeded by the red line usually regarded as the first distinctive sign of the disease. With the deposition of irritants dissolution of continuity continues, and the periosteum is laid bare and melts away. The points most liable to attack are those marked by greatest amount of gum tissue, or about peculiarly shaped, or irregularly placed teeth. The susceptibility to attack, and the progress of the disease, are influenced by the contour of the parts, the habits of the patient, the nature of the cause, etc. The direction and rapidity of the advance under different types of alveolus in breadth and depth, as attacking anterior or posterior surfaces or septums, and the aspect of the gum under these different conditions were carefully considered; also the influence of the mode of life of the patient, whether active or sedentary, temperate or otherwise. The environments are also a factor, as seen in the effects of the use of mercury by early settlers in the west, and of salt, as in the abnormal cravings caused by malarial poison, or the salt meat diet of sailors and laborers, the disease, in its typical form, being considered by the writer directly traceable to these sources of causation, and transmittible in hereditary form. Though acute ptyalism passes rapidly away, the effect upon the tissues is permanent and transmittible, the typical form of Pyorrhœa due to the use of mercury or of salt not being curable. This form does not appear till middle age, caries also being held in *statu quo* by this condition of the system, secretions, or tissues. When transmitted, it becomes apparent in childhood, and is very difficult of control, the bony structures melting away, though the gums hold their position for a time. When thus transmitted it appears independent of all local causes, as tartar, fungus, or any external irritant, a predisposition growing into dyscrasia. In the second or third generation it may be checked.

But one mode of treatment is of any avail, viz : Thorough removal of all deposits, cleansing from debris, washing with antiseptics, as Listerine, and leaving a clot of blood, nature's own remedy, to protect the parts. The removal of the deposit is often a work of great difficulty, and requires, upon the part of the operator, a steady hand, cool head, delicate sense of touch, ability to detect the difference between live and dead bone, etc. The necessary instruments were described, and the mode of manipulation. The paper closed with the suggestion of the following points for discussion :

1st. Are deposits of salivary calculus the cause of or pathognomonic of Pyorrhœa ?

2d. Is Pyorrhœa infectious ?

3d. Where the disease is common to a family, is it due to infection or heredity ?

4th. Is there a fungus peculiar to this disease ?

The paper elicited an interesting and spirited discussion.

*Dr. Walker*—Regretted the absence of Dr. Riggs, the pioneer in the scientific recognition and treatment of this disease, which has been the *bete noire* of the profession for many years. He fails in effecting cures by surgical operations alone, but thinks the disease requires concurrent local and systemic treatment.

*Dr. Reeve,* (N. C.)—It is most essential to know the cause of the disease. Pyorrhœa is rather a symptom than a disease *sui generis.* He considers that it has its inception in alcoholic stimulants, which increase the amount of uric acid eliminated. He has had the calculi analyzed, and found them to be almost pure uric acid. The deposits occur farthest from the direct influence of the heart's action, and in parts of low vitality. It is confined to males, or if existent in women, it is not until after puberty. The remedies required are those which decrease the elimination of uric acid, as atropine, digitalis, acetate of potash, cod liver oil, etc. Negroes never are attacked by it.

*Dr. Morgan,* (Tenn.)—According to my experience, negroes have it as frequently as white men, and females of all ages as often as men. I have, at the same time, treated a mother with a suckling babe, and her daughter fifteen years old. I know that for three generations no alcohol had been used in that family, yet Pyorrhœa had existed from grandfather down. It is highly commendable to analyze the deposits, and I am glad to learn of such investigations, but I have never before heard the disease attributed to uric acid. The general health is often broken down, and constitutional treatment is required. It is often confounded with scurvy, and indeed these are some points of resemblance.

*Dr. Taft,* (Cincinnati)—There is a great difference of opinion as to the origin and cause of Pyorrhœa. There is connected with it a condition of the system in which the tissues readily yield to irritants; there is a want of elimination. It occurs where the teeth are not properly exercised, and there is a consequent lack of nutrition. Imbibition of pus takes place, causing a degree of blood poisoning, or mild pyæmia. Systemic conditions cause the disease and are also caused by it. Local treatment is of no avail until the general conditions are rectified and the system toned up. In the treatment of this disease we should study principles rather than remedial agents.

*Dr. J. A. Robinson,* (Michigan)—In my old age I have given to the profession "The Robinson Remedy," a specific for this disease, which will secure results with one treatment, or in twenty-four hours, that would not seem possible. Employing it in the worst cases, the pockets will half close up in one treatment, and the gums hug the teeth after the second. A third application is never required. Of course all deposits must first be thoroughly removed, and loose teeth ligated. The remedy is carbolized potash; equal parts of crystals of carbolic acid and caustic potash. If they do not deliquesce, add enough water to make a creamy paste. If it crystallizes afterwards, heat the bottle and again liquify it. Apply it to one tooth at a time on a small rope of cotton dipped in the bottle, and laid on a napkin to absorb the surplus. Cut off the proper length for a tooth and apply it, leaving it in position only while applying a piece to the second tooth. He described a number of remarkable cures wrought with it.

*Dr. J. R. Walker*—Hopes that the Robinson remedy will do all that its originator claims for it. But different cases require different treatment. When one remedy fails we should try another. He has found the following very efficient, after thorough surgical treatment.

| | |
|---|---|
| Carbolic acid, | ʒj, |
| Tincture Iodine, | ʒj, |
| Glycerine, | ʒx. |

Mix well, and add six parts of Labarraque's solution of chlorinated soda. It becomes colorless and more volatile than water; then add ʒj to ij of the crystals of the chloride of zinc, when it coagulates and is strongly astringent. Apply this with the thin end of a toothpick, shaped like a spatula, that will reach into the pockets. In my hands it accomplishes almost what Dr. Robinson claims for his remedy. The dark-blue blood is changed to a lively red; granulations begin at the bottom, and the pockets fill up readily.

Dr. Morgan paid an elegant tribute to the memory of Dr. Prothro, deceased.

Adjourned to 7.30 P. M.

SECOND DAY—EVENING SESSION.

The President in the chair. The discussion of the paper on Pyorrhœa Alveolaris was resumed.

*Dr. Harlan,* (Chicago)—Dental practitioners in inland cities have comparatively few opportunities for studying the teeth of sailors on the high seas; but with them, as with the Irish laborer, the condition of the mouth is more likely to be due to the absence of all ideas of cleanliness than to the deprivation of vegetable food or the use of salt meats. They have salivary calculus, but seldom Pyorrhœa Alveolaris. Such theories carry their own refutation with them. The condition of the gums after ptyalism does not resemble Pyorrhœa. Mercurial diathesis may be transmitted, but acute ptyalism is not a hereditary disease. It was well said in the paper that escharotics should not be employed, but rather that germicides and antiseptics are beneficial. In mouth-breathers the palatal aspect is affected. Salivary calculus *per se* has nothing to do with the inception of Pyorrhœa Alveolaris. After removal of the calculus the peridental membrane may remain well attached, so that the probe cannot pass. Pyorrhœa deposits are not continuous; they form little islands. Deposits are more frequent upon the labial and buccal aspects than between the teeth. If they appear on the mesial or distal surfaces it is when the adjacent teeth have been extracted. Thorough removal of all deposits is absolutely essential. The edges of bone must be cut away and scraped or burred out. Failure in removing the debris is often the cause of a non-cure. To secure this it is sometimes necessary to make a transverse slit in the gum, exclude the saliva and inject peroxide of hydrogen in full strength. The blood-clot will furnish a protection, but it is not transformed tissue. The disease is a difficult one to treat, and the cases which get well under simple treatment are usually not pyorrhœa at all, but salivary calculus. We need something to destroy the bacteria. True Pyorrhœa is infectious. This he had demonstrated in one case by injecting pyorrhœal pus under healthy gum, and thus inoculating the disease. Instruments should be cleansed in preparations that will destroy the spores. We must beware of over-treatment, and allow nature a chance to do her work.

*Dr. Jas. S. Knapp,* (New Orleans)—Pyorrhœa is not a new disease. It is older than Dr. Riggs himself. It is not caused by tartar, for if this were the case, all who have tartar would have Pyorrhœa. It is the lack of boldness in operator and cowardice on the part of the patient that causes failure, by preventing the thorough removal of deposits. His first application is chloride of zinc; after a few moments this is washed out and followed with tincture of iodine. This may cause discoloration, but it is only temporary. Dr. Atkinson claims that lost alveoli can be reproduced, but this is absurd. Perhaps it is not impossible, but it is at least doubtful.

*Dr. J. R. Walker*—Dr. Riggs was the first to apply the surgeon's maxim, " cut beyond the dead line." Modern success in treatment is largely due to him.

*Dr. A. O. Rawls*—My views have been misconstrued. I contend that when the process is broken down and denuded of its membrane, restoration is not possible. Inflammatory action may be checked, but the conditions are still present. Nutrition is resumed in the soft tissues, but not when the connection of the tissue with the tooth is broken. I have never known a typical case that could not be traced to mercury or salt. I have made it my business to hunt up and study the cases of sailors and laborers in this connection, and invariably find these conditions present. The theory may be wrong, but a strange coincidence certainly exists. I do not contend that mercury will invariably produce these conditions, but that when these conditions do exist they are caused by mercury. Mercury certainly does produce conditions that render possible this peculiar disease, so difficult of cure. Fungi and bacteria follow the destruction of tissue; they are the resultants, not the starting points. Restoration of tissues is the essential thing, not the killing of vegetable growths. Remove dead bone and debris, and give protection to the parts. This is all we can do.

(An interesting and animated *tri*-alogue ensued on the nature of heredity, with Dr. Rawls on one side, and Drs. Taft and Morgan on the other, the former maintaining that heredity in the tissues resulted from an impression made upon the molecules, the latter insisting that heredity was of typical peculiarities only.)

*Dr. J. R. Walker*—The more thoroughly we study causes, the better we can treat results. Dr. Rawls' observations led him to consider mercury and salt as prime factors of causation. My own observations point to *butter* or *grease*. I desire to ask Dr. Rawls to look for the results of fried grease or cooked butter. Deposition of calculus indicates a misdirection of nutritive elements.

*Dr. Fountain,* (Texas)—Pyorrhœa is very prevalent in the Brazos bottoms, where malaria prevails and mercury is much used. Most of my Pyorrhœa patients say they have been salivated.

*Dr. Patrick,* (Belleville, Ills.)—A concatenation of circumstances bringing about certain results may be considered as accounting for those results. That accidental physical peculiarities are transmitted, there is no doubt. I know of one family where, for several generations, even to first cousins, the two first fingers are missing. Why? We do not know. It is so; that is all. The fact is undoubted, but the reason is in darkness.

*Dr. Robinson*—This is especially the case with the teeth. I know of one family where, for four generations, there are no superior lateral incisors.

*Dr. Teague,* (S. C.)—Cited the case of a certain family in South Carolina, the members of which have been edentulous for generations.

Numerous other illustrations of the principle of heredity were related by members, after which the discussion was closed

Dr. J. A. Robinson, of Jackson, Mich., read a paper, entitled "How to Increase Your Business," a paper replete with sound advice, based on the results of nearly a half century's experience in the dental profession. Starting with the suggestion that to outstrip others in the race for fame and fortune we must do what others are not doing, the first piece of advice given was to read all the journals, in order to know what has been done, what is being done, and what is left undone.

The ratio of success will be in proportion to the permanence of the work done.

The salvation of the natural teeth should be the principal object, artificial substitutes serving only to make things bearable, and as a last resort.

Among the fields as yet comparatively uncultivated by the average dentist, and open to the young man, he named the study and treatment of diseased teeth and gums, the setting of artificial crowns, correcting irregularities, etc.

As a means of gaining increased patronage, he especially considered the cultivation of the fraternal feeling in the profession, our confidence in each other attracting the confidence of the laity. It is not enough to believe in our own abilities; we must make the world believe also. The way to do this is by modest advertising, and the most dignified means is the publication of information for the people as to the value of the teeth, the rates of compensation being in proportion to the understanding of the value of the teeth, and of our services.

The hour for adjournment having arrived, discussion of the paper was deferred until the next session. Thursday being dentists' day at the World's Exposition, and Friday devoted to clinics and the exhibition of appliances, the Association adjourned until Friday evening.

